# Danger of Excess of Economy in Child Welfare Work

**Published:** 1916-04-08

**Authors:** 


### THE VALUE OF CHILD LIFE.

### Danger of Excess of Economy in Child Welfare Work.

The Registrar-General's latest return of marriages, births, and deaths in England and Wales forcibly illustrates the effect of the war on social conditions. During the third quarter of 1915 there were more marriages than in any corresponding period since civil registration was established, and in the last quarter the birth-rate fell to a figure never before recorded. The extent by which births exceeded deaths is stated as follows:—

| | | | | | |
|---|---|---|---|---|---|
| 1912 | ... | 87,995 | 1914 | ... | 77,394 |
| 1913 | ... | 89,045 | 1915 | ... | 46,368 |

For the whole of 1915 the excess was 252,201, as against an average annual increase of 378,360 in the preceding five years. The total births for the fourth quarter were 183,445, or a decrease of 24,831 on the total for the corresponding quarter of 1914 and 22,845 on that of 1913. The infant mortality rate was 114 per 1,000, or 5 per 1,000 below the decennial average.

The total of excess of births over deaths has been falling for some time, but the extraordinary results here quoted must bring into prominence certain problems in the minds of all interested in social matters.

Foremost must come contemplation of the fact that, as many of the flower of our manhood have been and will be killed or injured in the war, the value of child life cannot be overestimated at the present time. Further, we must be prepared, when the war is over and hostilities cease under the most favourable terms for the nation, to face questions of employment, of emigration, of financial conditions preventing marriage or restricting the rearing of families, which will have a marked effect upon the number of children born and the efficiency of the young generally. The State, which has never encouraged a high birth-rate, and has taxed indiscriminately the large and the small family, will possibly be forced to undertake the solution of the difficulty in such a way as will cause to sink into comparative insignificance any democratic legislation of the last decade, and by setting up preferential conditions will coax or force potential fathers and mothers from the anti-patriotic policy of negation.

In searching for a brighter side of the picture we notice that the marriage-rate has been higher. The cause of this is probably twofold, the ephemeral prosperity of the civilian population and the "war-marriage," a union often un-eugenic because of the hasty and perhaps careless way in which the parties are brought together. The first group of marriages will produce children born in times of financial family prosperity who, probably during early years, will share the effects of a return to much humbler conditions. The issue of the "war-marriage" group will come into the world in unsettled homes and often under far from favourable circumstances. Thus in respect of the mass of the population we cannot hope for a high health standard in children born of unions contracted under war conditions.

Particularly is this a time when a zealous eye must be directed to any movement which may bring about the false economy of reducing necessary activities in the direction of Child Welfare work; now more than ever any carefully prepared scheme having for its object the well-being of bearing women and their offspring must receive due consideration.

Work for Children in Bradford.

It will be remembered that in The Hospital recent reference was made to Child Welfare work in Bradford in two connections. The first was in respect of the failure, as promised, of the municipality to increase the salary of Dr. Helen Campbell, the chief medical officer of the Bradford Infants' Clinic, her resignation and subsequent withdrawal of her resignation "in the interests of the cause of infant child life in Bradford." The other connection was with regard to the statement made by Dr. H. G. Campbell, the senior honorary physician of the Bradford Royal Infirmary, as to the cost, degree of success, and extent of work done for children at the infirmary as compared with the municipal organisation.

Dr. Campbell, judging by the report of his speech, appeared to attach a great deal of importance to the fact that the infantile mortality rate (children dying in the first twelve months of life) in Bradford had risen instead of fallen since the opening of the municipal Infants' Clinic in 1912. He stated the rate per 1,000 births since 1909 to be as follows: 1909, 116; 1910, 127; 1911, 140; 1912, 99; 1913, 128; 1914, 122. It would be a matter for regret if adverse criticism is lightly passed, during the first three or four years of its career, on any institution carefully inaugurated as the result of public needs; but when that criticism is based on the want of effect of one part of the labours of an institution of limited capacity upon the mass of the population of a great industrial city the reasoning of the critic must be followed with some care.

Before proceeding to other questions, let us examine the vital statistics as quoted by Dr. Campbell. It is a truism that many of the children dying in one year are those that were born in the preceding year, and it is well known that a high death-rate in one year often means a low one in the following year. For instance, the year 1911 had a very hot summer, and deaths amongst very young children were numerous all over the country; in 1912, as one would expect, the rate fell considerably. It would, therefore, be unwise to assume anything from the rise in infantile mortality between 1912 and 1914. There may be an equally good reason for the exceptionally low rate of 1909, and the comparison of that year with a selected subsequent year may be quite likely vitiated by circumstances. The object of these remarks is to show that single-year comparisons are of little value for the purpose; periods must be taken of sufficient duration to cover quite a variety of causal circumstances.

In any event, why should a case be prepared on these statistics against an institution which is building up effort not mainly for present effect, not only in the interests of children in the first year of life, but of educational and administrative value which is designed also to influence the well-being of unborn children, of children from birth to puberty, and to improve in many respects the social conditions of families generally? And if we condemn on this particular phase of vital statistics an institution working under the principle of prevention, must we let go scot-free Dr. Campbell's hospital which is devoted chiefly to cure?

A study of the Local Government Board returns teaches us that a great saving of child life has been effected in the last few years, and that this is considered by the Department to be the result of improved sanitary and housing conditions, of more efficient municipal and domestic cleanliness, of education in hygiene, of the increased sobriety of the population, and the wide-spread awakening to the national importance of child mortality, together with concentration of efforts of Child Welfare work such as had never previously occurred.

In this great social work hospitals have given efficient help, but all parts of the main scheme have limitations, and in criticising one organisation or comparing it with another it must clearly be understood what particular service each renders and how far there is possibility of overlapping and consequent want of economy in money and in human effort. It may, therefore, be our duty to return to this subject again, entering more fully into the respective work as regards the welfare of children and bearing mothers, of the Bradford Royal Infirmary as representing hospitals, and of the Bradford Maternity and Child Welfare scheme as representing specialised social effort in the saving of child life.

